# Cyberbullying: Common Predictors to Cyber-Victimisation and Bystanding

**DOI:** 10.3390/ijerph192315750

**Published:** 2022-11-26

**Authors:** Daniel Lloret-Irles, Víctor Cabrera-Perona, Sonia Tirado-González, José V. Segura-Heras

**Affiliations:** 1 Department of Health Psychology, Miguel Hernández University, 03202 Elche, Spain; 2 I.U. Operations Research Center, Miguel Hernández University, 03202 Elche, Spain

**Keywords:** cyberbullying, risk factors, cyber-victimisation, bystanders, prevention

## Abstract

Cyberbullying has increased worryingly in the last decade, becoming a mental health problem in adolescence. Research usually focuses on cyber-bullies or cyber-victims, overlooking that these roles may overlap (e.g., cyber-victim-bystander). Aim: To identify possible common predictors to cyber-victimisation and bystanding. Sample: The study sample consisted in 560 students, 12–15 years old, 47.5% female. Method: Canonical correlation, examining linear relationship between a group of X variables, and a group of Y variables. Main results and conclusions: Two canonical varieties were built (Cor (U_1_,V_1_) = 0.442; Cor (U_2_,V_2_) = 0.270). Minors with high scores in cyber-victimisation (r = −0.888) and bystanding (r = −0.902) would have more favourable attitude towards violence, greater number of contacts on social networks/messaging and greater attention to emotions. The second variety discriminates minors with high cyber-victimisation score, but low observation and would relate to low attitudes towards violence and contacts on social networks/messaging, together with high scores in parental monitoring. Results suggest the possible overlapping of roles and how cyber-victimisation and bystanding share predictive factors.

## 1. Introduction

Along with the undeniable benefits and opportunities of ICLTs (Information, Communication and Leisure Technologies), new ways of interaction among minors also provide new methods for peer victimisation, for example, cyberbullying. The term cyberbullying defines any form of intimidation or intentional harassment, repeatedly and continuously among equals through electronic devices, taking place in a school environment or outside of it [1,2]. In Spain, between 20% and 50% of school children between 12 and 17 years old, report having had contact with cyberbullying at some point, either as cyber-victims, cyber-aggressors, or bystanders [3,4]. In addition, between 2.9% and 5.5% declared that they have acted as cyber-aggressors over a maintained period of time, while between 2–7% report maintained and severe cyber-victimisation [3,4]. However, these figures could be higher since in many cases the cyber-victims never come forward or inform of what happened [5]. There has also been an increase in cases in the last decade [6]. Thus, cyberbullying is a growing concern in social and school environment, which has a strong psychosocial impact on all the actors involved, but especially on cyber-victims, who may manifest problems such as depression, low self-esteem, social isolation, poor academic performance, or social anxiety, among others [2,3,6].

### 1.1. Protection and Risk Factors

Different models such as (a) Cyclic Process Model [7] and (b) Barlett and Gentile Cyberbullying Model [8] hypothesise that the different figures involved in the phenomenon could become other figures (e.g., cyber-victims become cyber-agressors). This could be the effect mediated by the emotional regulation that minors are capable of carrying out, meanwhile, attitudes towards cyberbullying lead to perpetration, respectively. In this sense, it would be pertinent to ask if the figure of passive bystander could be an intermediate step in this dyad. Thus, there is an interest to better know and evaluate different factors that can contribute to predict the different figures involved in cyberbullying, such as contextual factors, factors related to the use of ICLTs, and individual and family factors. 

Regarding contextual factors, it has been suggested that a greater frequency of daily connection, often joined by less privacy (e.g., providing personal data, adding strangers, publishing intimate photos) would expose minors to a greater risk of cyber-victimisation [2,9,10,11,12]. On the contrary, contemplating privacy norms and using protection and security software have been related to a lower probability of cyber-victimisation [11]. In regard to individual factors, some studies suggest that a positive attitude towards violence is positively related to higher levels of cyber-aggression, specifically beliefs that consider aggression to be an acceptable method for conflict resolution and relationship management [11].

Likewise, low empathy has been found to be linked to higher rates of cyber-aggressive behaviours [13], both in cyber-aggressors and cyber-victims [14], with cyber-aggressors having less affective empathy than cognitive empathy [15]. Finally, emotional self-regulation is a usual component of non-specific prevention programs and emotional education. Therefore, it is proposed that greater competence and abilities in recognizing one’s own emotions, and capacity to have emotional clarity and regulation would be a non-specific protective factor for risky and violent behaviour [16], and specifically in the area of ICLTs could be related to the daily frequency of connection, behaviours related to privacy, as well regulating possible consequences of cyber-victimisation. However, it has been poorly evaluated in cyberbullying, where for example cyber-aggressors have been referred to having adequate emotional self-regulation [17]. Nevertheless, studies that assess emotional self-regulation in cyber-victims or bystanders are scarce, suggesting that poorer emotional regulation would be related to greater cyber-victimisation [7]. With reference to family factors, some studies have shown that parental control, monitoring, and management of minors’ online behaviour can act both as a protection or risk factor, as it is inversely related to cases of cyber-victimisation [10,11,12] with higher degrees of cyber-victimisation being associated with aspects of a poor family adjustment [18].

### 1.2. The Role of the Bystander

Undeniably, online interactions among adolescents have a large audience in virtual environments. Bystanders can reconfigure the environment where cyberbullying takes place, (e.g., supporting the cyber-victim directly or indirectly, denouncing or extinguishing the cyber-aggression, breaking the chain of re-victimisation, etc.) [6,10]. Cyberbullying is maintained by reinforcing the cyber-aggressor, resending images, or being passive in an aggression [19]. Bystanders have been found to have lower empathy [20], lower pro-social behaviour [21], and a positive attitude and normative beliefs about aggression and violence [22]. It is reported that between 65% and 74% of students have witnessed some form of cyberbullying behaviour at least once, while 7–11% have done so frequently [3,23].

However, research usually focuses on one type of role (e.g., cyber-victim, cyber-aggressor or bystander), or a cyber-victim-cyber-aggressor dyad [2,12], overlooking the fact that on many occasions these roles could overlap each other. Recent studies show how cyber-victims most frequently witnessed episodes of cyberbullying in others and/or were also cyber-aggressors [12]. Addressing this overlapping of roles would add a better understanding of the phenomenon and allow for fine-tuning preventive interventions aimed at large groups of minors, recognising the commonality of risk factors and helping to get bystanders to act towards stopping the episode and providing support for the victims [5,6,19].

Despite the remarkable increase in research in this field, there are still scarce studies that have taken into account risk and protection factors in the area of cyberbullying and included parental regulation of ICLTs, or emotional self-regulation as protective factors. Nor, to our knowledge, has it been investigated whether the same risk and protection factors could also be related to bystanders. Due to everything mentioned above, this study aims to (a) describe levels of cyber-victimisation and observation of cyberbullying behaviours in a sample of compulsory secondary education students, (b) identify possible psychosocial predictors of cyber-victimisation and bystanding of cyberbullying behaviours, and (c) explore the relationships between possible predictive factors common both to cyber-victimisation and bystanding. Regarding objective (a), we expect to find a direct relationship between cyber-victimisation and bystanding (Hypothesis 1); regarding objectives (b) and (c), we expect to find attitudes towards violence, empathy, emotional self-regulation, parental regulation of ICLTs, and behaviours regarding privacy as shared predictors of cyber-victimisation and bystanding (Hypothesis 2).

## 2. Materials and Methods

### 2.1. Participants

Questionnaires were administered to 605 students belonging to Compulsory Secondary Education (CSE) Spanish schools. After removing 45 cases due to inconclusive or random response, the final sample was 560 cases. Mean age was 13.34 years (SD = 0.90; Range: 12–15), 52.5% were males. Sample description can be found in Table 1 and Table 2. 

### 2.2. Procedure

The study was approved by the regional education authorities and the school board, and parental consent was obtained. Schools were selected by convenience according to geographical and social criteria with a ratio of two centres per town. All classes from each educational were eligible. The adolescents participated voluntarily after having been informed of the purpose of the study by researchers. No inclusion criteria were used. The questionnaires were self-administered collectively and anonymously, under the supervision of a psychologist, who resolved possible comprehension doubts. Completion time ranged from 30 to 40 min.

### 2.3. Variables and Instruments

Information about daily connection frequency and number of friends or contacts on social network was directly asked with two questions: “*How often do you connect to your social network*?” and “*How many friends or contacts do you have on your social network?*”.

*Cyber-victimization and observation behaviours of cyberbullying* were assessed with the cyberbullying questionnaire (CCB) [24]. In this study, only the subscales of cyber-victimisation and observation behaviours were used. Each of these subscales assesses 15 cyberbullying behaviours in the past year, to be answered on a 4-alternative Likert scale (1 = Never, 4 = Always). An example of item for cyber-victimisation subscale is: “*Has anyone made offensive and insulting calls to you* via *mobile or Internet?*”. An example of item for observation subscale is: “*Have you seen others making offensive and insulting calls* via *mobile or Internet?*”. Higher scores indicate a greater presence of cyber-victimisation and greater bystanding of cyberbullying behaviours. In the present study, the internal consistency of both subscales was Cronbach’s α = 0.90.

*Online Privacy Attitude and Behaviour was rated with the Online Privacy Attitude and Behaviour Scale* (EPO-20) [25] which consists of a 20-items scale organised in two dimensions: Attitude towards privacy and Privacy risk behaviours. An example of item is “*I accept most friendship invitations on social networks*”. A higher score reflects a greater degree of privacy on the minor’s behalf (range of response: 20–100). In this study, the internal consistency of the scale was α = 0.69.

*Attitudes towards violence in school context* were evaluated with the Scale of attitudes towards violence in an educational context (CAHV-25) [26], which consist in 25-item distributed into 4 factors: Violence as a form of entertainment; Violence to improve self-esteem; Violence to handle problems and relationships, and Violence perceived as legitimate. An example of item is: “*I find it fun to mess with some acquaintances*”. Higher scores indicate positive attitudes towards violence. The internal consistency of the scale for this study was α = 0.92

*Empathy* was assessed with the Basic Empathy Scale [27] of 9 items distributed in two subscales: affective empathy and cognitive empathy. An example of item is: “*I get sad when I see people crying*”. Higher scores indicate greater empathy. In this study, the internal consistency of the scale was α = 0.85.

*Emotional self-regulation* was measured with the Trait Meta-Mood Scale (TMMS-24) [28,29] compounded by 24 items distributed in three dimensions: (a) attention to emotions, (b) emotional clarity and comprehension, and (c) emotional repair-regulation. Dimension scores were used, since the total score is not recommended by the authors. An example of an item is: “*I pay close attention to how I feel*”. Higher scores indicate a greater degree of the evaluated dimension. For this study, the internal consistency for each dimension was: (a) attention and perception of emotions: α = 0.89, (b) emotional clarity and emotional comprehension: α = 0.84, and (c) emotional repair-regulation: α = 0.85. The last four scales are answered on a five-alternative Likert scale of agreement (1 = Strongly disagree–5 = Strongly agree).

*Parental control and mediation of screen use* was assessed with the EMP-TIC Scale [30], which includes 29 Likert-type items (0 = never, 4 = whenever possible, range = 0–116), and evaluates five dimensions of parental mediation: (a) restrictive mediation, (b) active mediation, (c) monitoring, and (d) monitoring software de) co-use. Total scores of each scale are obtained. An example of item is: “*How often your parents determine how long you can go online each day?*”. Higher scores represent a higher parental mediation. For this study, the internal consistency of the scale was α = 0.92.

### 2.4. Data Analysis

The tabulation and analysis of the data was carried out using the statistical software R. The quantitative variables have been summarised by the mean, the standard deviation, and the observed range of values, while for the categorical variables, recounts and percentages have been used. The Chi square test was used to measure the association between the responses to each item of the cyber-victimisation and observation scales. The Pearson linear correlation coefficient was calculated to measure the association between the variables. In all cases, a significance level of *p* < 0.05 was used.

A Canonical Correlation Analysis was carried out to assess the level of association of the two groups of variables [31,32]. For this analysis, we used only the variables that have shown a significant correlation with the outcome variables. The canonical analysis is an extension of the multiple regression analysis in cases of more than one continuous dependent variable. In addition, the canonical correlation identifies the optimal structure or dimensionality of each set of variables, which maximises the relationship between the sets of dependent and independent variables (we work with standardised variables to avoid scale problems):

- Outcome variables (X): cyber-victimisation, observation
- Predictors variables (Y): Age, daily frequency of connection, contacts in social networks/messaging, parental mediation-monitoring, parental mediation-co-use, emotional self-regulation-attention to emotions, attitude towards violence as a form of fun, attitude towards violence-improvement of self-esteem, attitude toward violence-management of problems, attitude towards violence considered legitimate.

The main objective is to find pairs of new variables U = a_1_ × 1 + a_2_ × 2 and V = b_1_Y_1_ + b_2_Y_2_ + … + b_10_Y_10_, which are a linear combination of the original values, with the property that their correlation is the maximum possible. We call U_1_ the first canonical variable of the set X, V_1_ the first canonical variable of the set Y, and the resulting correlation between them, the first canonical correlation.

The maximum number of pairs of canonical variables that we can construct is the minimum between the number of variables in set X (2) and the number of variables in set Y (10). In this case, we will have two pairs of canonical variables (U_1_, V_1_) and (U_2_, V_2_). The associated canonical correlations decrease as the number of canonical correlations increases. We use Wilks’ lambda, using Rao’s F-approximation, to test whether these correlations are significantly different from zero.

## 3. Results

### 3.1. Prevalence of Cyber-Victimisation and Bystanding

The prevalence of cyber-victimisation in terms of occasional cyberbullying varies between 4.1% (recorded aggressions) and 25.6% (offensive messages through messaging or Internet), depending on the type of behaviour. Frequent cyberbullying varies between 1.1% (death threats by mobile or internet) and 6.1% (anonymous calls with the intention of frightening). Occasional bystanding ranges from 2.2% (death threats via mobile or internet), 11.1% (sexual harassment through mobile or internet), 19.9% (others making offensive and insulting messages), and 31.4% (other receiving offensive and insulting messages).

Table 3 shows differences between bystanding and cyber-victimisation. When the minor does not act as a bystander, the prevalence of occasional cyber-victimisation ranges between 1.5–12.9%, rising to between 8.5–33.7% for occasional bystanders and to 13.6–44.1% for frequent ones, depending on the modality. Regarding frequent cyber-victimisation, this oscillates between 0.3–1.9% for no-bystanding, 1–7% when occasionally bystanding, and 5.3–25% when the minor refers to high observation (Table 3).

### 3.2. Canonical Correlations 

In Table 4, the correlations between the groups of variables X and Y can be observed.

The correlations observed between each pair of canonical varieties are 0.442 (U1 and V1) and 0.270 (U2 and V2), respectively. By definition, U1 and U2 are uncorrelated, as are V1 and V2. U1 is characterised as a general average of cyber-victimisation and observation, given that both variables have a high correlation with U1 in the negative sense (r = −0.888 and r = −0.902, respectively). The second canonical variable, U2, discriminates between both variables, cyber-victimisation positively (direct relation, r = 0.459) and observation negatively (inverse relationship, r = −0.431) (Table 5).

Regarding V1, it can be observed that the most correlated variables are violence as a form of problem management (r = −0.684), violence as an improvement of self-esteem (r =−0.680), violence as a form of fun (r =−0.608), and to a lesser extent, daily connection frequency (r = −0.476), contacts on social networks/messaging (r = −0.461), emotional self-regulation-subscale of attention to emotions (r =−0.430). and violence considered as legitimate (r = −0.410). The correlation of the rest of variables can be considered low (r < 0.40). Regarding V2, we can highlight the relationship with parental monitoring (r = 0.668) and violence considered legitimate (r = −0.562), and to a lesser extent with contacts on social networks/messaging (r = −0.476). The rest of the correlations can be considered low (r < 0.40) (Table 6).

**Figure 1:**
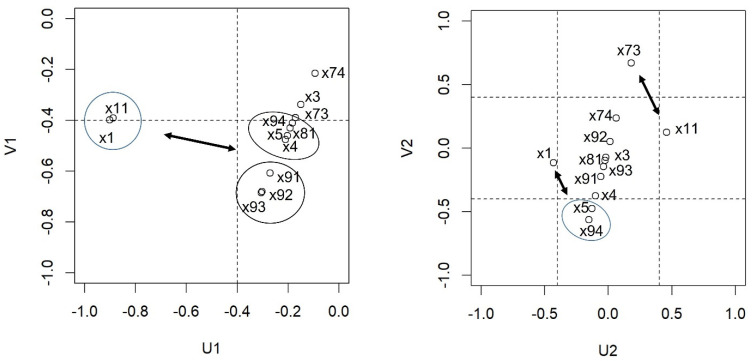
Correlations between the studied variables and the canonical varieties. First variety U1. Second variety U2.

As mentioned above, the correlation between U1 and V1 is 0.442, therefore, high values of U1 are associated with high values of V1 and vice versa. This leads us to characterise individuals who have higher scores in cyber-victimisation and observation, as people who will present high scores in violence as a form of entertainment, to improve self-esteem, manage problems, and to a lesser extent, daily frequency of connection, contacts on social networks/messaging, emotional self-regulation, subscale of attention to emotions, and violence considered as legitimate (Figure 1). 

On the other hand, the correlation between U2 and V2 is 0.270. The variable U2 discriminates between the variables cyber-victimisation and observation. Ergo, individuals with higher values in cyber-victimisation and lower scores in observation will present high values in U2. In the same way, V2 discriminates between parental monitoring against violence considered as legitimate and contacts on social networks/messaging. This second canonical variety associates low scores between observation, violence considered as legitimate, and contacts on social networks/messaging, with high scores in cyber-victimisation and parental monitoring (Table 5 and Figure 1: Correlations between the studied variables and the canonical varieties The first variety being U1, and the second variety being U2).

## 4. Discussion

This study aimed to describe the prevalence of cyber-victimisation and bystanding of cyberbullying behaviours among adolescents and to identify possible common predictors to both roles. The prevalence of cyber-victimisation in our sample are in line with previous studies in our country, both in terms of occasional cyberbullying (4.1–25.6%) and frequent cyberbullying (1.1–6.1%), depending on the modality [3,24]. Regarding the observation, our work shows the bystanding of at least one episode of cyberbullying in 15.5–51.3%, depending on the type of behaviour, while other studies show somewhat higher values (35.4–65%) [3,33]. Differences could be explained by the methodology (e.g., modalities of cyber-bullying taken into account, period of time covered by the items, etc.). In addition, results show a direct relationship between cyber-victimisation and bystanding (Hypothesis 1) (Table 3). In general, the high degree of cyber-victimisation among non-observers is almost residual (0.3–1.9%). However, within most frequent bystanders, the figures increase to 5.3–25%.

### 4.1. Canonical Correlations: Predictors and Relationships 

In our analysis, we tested the fit of several models. The inclusion of the variable bystanding increased explained variance, although excluded other variables from the model, which could mean that it might moderate other predictable variables (Hypothesis 2).

Results show greater explanatory power for profiles of minors that have a high degree of bystanding behaviours and cyber-victimisation, but feebly explains the profile of cyber-victims that do not refer to bystanding. It suggests that adolescents with high scores on cyber-victimisation and bystanding would also have a higher favourable attitude towards violence (as a form of entertainment, as an improvement of self-esteem, as a form of management of their problems), together with a greater attention to their emotions. In addition, minors with higher cyber-victimisation, but with a low degree of observation of cyberbullying behaviours, would present a lower degree of positive attitudes toward violence (considered legitimate), as well as fewer contacts on social networks/messaging, and higher levels of parental monitoring. This can be considered evidence of the relationship between attitude toward violence and a higher degree of both cyber-victimisation and bystanding of cyberbullying (Hypothesis 2). Other studies have shown that not only cyber-aggressors, but also bystanders, presented greater use of aggressive strategies to resolve interpersonal conflicts [8,22]. Our results coincide with this proposal, suggesting that in many cases, the roles overlap [12]. In fact, the complexity of interactions between cyber-aggressors, cyber-victims, and the possibility of playing multiple roles simultaneously has been referenced as a line of research [11,34]. Thus, in our sample, favourable attitudes to violence and cyberbullying would be a common element for bystanders, but also for cyber-victims, who could legitimise acts of cyberbullying and frame the virtual interactions of minors, establishing “rules of online behaviour” among a certain group of minors.

Emotional self-regulation is related to higher cyber-victimisation (Hypothesis 2). This data makes sense when we consider other studies in which an excessive score of emotional self-regulation is associated with greater neuroticism and worse psychological adjustment [35,36]. Likewise, minors who have witnessed cyber-victimisation have been found to have greater degrees of neuroticism and worse psychological adjustment than minors who have not been victims [3]. Thus, we may be looking at an indirect measure of neuroticism, which in this study would be shared by minors who report greater cyber-victimisation and who are also observers of a higher number of episodes of cyberbullying. These minors would pay more attention and concern to their moods, but would not put in place shock-absorbing mechanisms (evaluated by the other two subscales of the instrument: emotional clarity and emotional repair). In other words, these minors would have worse emotional regulation, supporting postulates of Cyclic Process Model [7]. In this sense, another study [37] on the subject of traditional bullying shows that victims exhibited a similar pattern, having higher attention to emotions, but low scores in clarity and emotional repair. To determine if this greater attention and perception of emotions is a product or antecedent of the greater cyber-victimisation and observation of cyberbullying is something that future longitudinal studies should attend to. 

Regarding family factors, contrary to what was described by other studies, parental control did not behave as a protective factor [10,11] (Hypothesis 2). In the line of another study, where a greater parental control predicted a higher degree of cyber-victimisation [33], our analysis show that stronger parental monitoring is related to a higher degree of cyber-victimisation along with a low degree of bystanding. Being cross-sectional studies, a possible explanation could be that this is due to ineffective attempts of parental control and monitoring, or that parents increase their parental control monitoring once their minors have given some sign of suffering cyber-victimisation. Our analysis prevents clarifications in this regard.

In relation to contextual factors, contrary to what was expected and observed by other authors [11], in our sample, we put into place behaviours regarding privacy or not was not related to cyber-victimisation or the bystanding of episodes of cyberbullying (Hypothesis 2). Differences also were not found between the group with the highest cyber -victimisation and the rest of the sample regarding privacy practices. In line with the previous result, it could be that those adolescents who reported greater cybervictimisation had implemented compensatory privacy practices once they had suffered cybervictimisation, possibly as a result of parental mediation.

### 4.2. Practical Implications 

In summary, our results support the presence of an overlap between the roles of bystanders and cyber-victims in cyberbullying, showing a possible profile of minors that share favourable attitudes towards aggressive behaviours and inadequate emotional regulation. These results contribute useful information for practical applications such as preventive school or familiar interventions. In this regard, it would be reasonable to include in universal prevention programs components aimed at changing attitudes towards violence and towards cyberbullying, as well as not only focusing on cyber-aggressors but on all adolescents, given the fact that they can all be a predictors of cyber–victimisation, due to the high level of bystanding. Decreasing positive attitudes towards violence and making minors aware that cyberbullying has negative consequences on cyber-victims, are basic steps for bystanders to shift from a passive to an active style and contribute to stopping cyberbullying [38,39]. Besides, it could be an influential component for fostering constructive bystander active interventions [6]. 

In addition, it is necessary to incorporate components of emotional education to compensate minors’ inadequate attention to their emotions [35]. More effective emotional regulation could reduce the cyclical process that turns cyber-victims and bystanders into cyber-aggressors later. Thus, in the preventive school setting, it would be of interest for practitioners to pay attention to high scores on emotional regulation scales. On the other hand, and in reference to cyber-victimisation, the data obtained also stresses the importance of taking into account parent’s involvement in prevention programs, once the sense of the relationships of the strategies, put in motion by them, is confirmed. Thus, other studies support our findings, noting that adolescents who perceive lower levels of parental control are involved in cases of cyberbullying [2,12].

### 4.3. Limitations of the Study and Future Lines of Research 

Our proposals must be interpreted taking into account the usual limitations of a cross-sectional study based on self-reports. Therefore, our results only allow us to suggest relationships. Besides, not assessing cyber-aggression prevents us from triangulating the three main roles of the phenomenon. However, the interest of this study was to consider the observers as key characters. In this sense, the role of bystanders is complex and ranges from assistants of cyber-bullies to defenders of the cyber-victims [21,40]. Although our study contributes the reduction of aggressive attitudes as a possible factor for the mobilization of bystanders, this makes it necessary to accept the results with caution in future studies. Besides, it would be advisable for future research to consider these aspects, seeking repeated or longitudinal measures, as well as delving into possible differences that address sociodemographic variables such as sex and age. Additionally, to carry out analysis that would allow to establish profiles with the aim of differentiating subgroups that require prevention or specific intervention (e.g., only cyber-victims, but also observers and/or aggressors, etc.) would be of interest. Likewise, it would be of interest to consider variables related to online risk behaviours (e.g., privacy, parental mediation and control). 

## 5. Conclusions

With the aim of contributing to preventive interventions in educational contexts, the present study provides evidence regarding the possible overlapping of roles in the cyberbullying phenomenon. The high correlation between cyber-victimisation and bystanding, could be explained by the fact that they share predictive factors. Results show greater explanatory power for profiles of minors that have a high degree of bystanding behaviours and cyber-victimisation, but feebly explains the profile of cyber-victims that do not refer to bystanding. The findings suggest the relevance of paying attention to the figure of the bystander, not only as a non-participating observer, but also as an audience involved in cyberbullying episodes. Thus, results underline the importance of the audience in this phenomenon and suggest that preventive interventions should address attitudes towards violence and emotional regulation among all adolescents. In this sense, any preventive intervention that tries to mobilise bystanders should take into account (a) the possible violent attitudes present in the observer and (b) the emotional recognition that as cyber-victims they may have.

## Figures and Tables

**Table 1:** Description of the sample. Qualitative variables.

Variable	%	n
Sex		
Female	47.5	266
Male	52.5	294
Age		
12	12.5	70
13	54.5	305
14	21.4	120
15	11.6	65
Academic year		
1 °CSE	22.5	126
2 °CSE	77.5	434
Frequency of daily connection		
Continuously	23.4	131
1–2 h/day	28.0	157
3–4 h/day	14.1	79
5–6 h/day	7.0	39
Occasionally	21.4	120
No connection	6.1	34
Contacts on social networks/messaging		
No social networks	3.4	19
Less than 10	2.5	14
10 and 50	16.6	93
50 and 100	20.7	116
100 and 300	31.6	177
Over 300	25.2	141

**Table 2:** Description of the sample. Quantitative variables.

Variable	M	DT	Range
Age	13.34	0.90	12–16
Privacy			
Privacy behaviours	37.05	5.58	11–55
Attitudes towards privacy	31.86	4.77	9–45
Total scale	68.91	8.16	20–100
Parental regulation			
Active regulation	16.58	9.90	0–36
Restrictive regulation	8.16	5.36	0–20
Parental monitoring	7.04	6.88	0–40
Co-use	6.97	3.90	0–20
Total scale	38.74	20.72	0–116
Emotional self-regulation			
Attention to emotions	22.07	7.83	8–40
Emotional clarity and comprehension	23.79	7.23	8–40
Emotional regulation and management	25.77	7.76	8–40
Attitude towards violence in a school context			
FI: Violence as a form of fun	13.20	5.53	7–35
FII: Violence to improve self-esteem	8.15	3.87	5–25
FIII: Violence to handle problems and manage relationships	10.25	4.45	6–30
FIV: Violence perceived as legitimate	19.24	5.92	7–35
Total scale	50.84	17.20	25–125
Empathy			
Affective empathy	13.30	4.18	4–20
Cognitive empathy	19.30	4.11	5–25
Total scale	32.60	7.41	9–45

**Table 3:** Cyberbullying behaviours. Cyber-victimisation and observation. Chi Square Test (% row (N)).

		Cyber-Victimization			
Item.	Observer	Never	Sometimes	Often-Always	*p*-Value
1. Have you seen how other have sent offensive and insulting messages via mobile or Internet?	Never	86.4 (235)	12.9 (35)	0.7 (2)	<0.001
	Sometimes	63.4 (111)	33.7 (59)	2.9 (5)	
	Often-Always	36.9 (41)	44.1 (49)	18.9 (21)	
2. Have you seen others making offensive and insulting calls via mobile, Internet, Skype, etc.?	Never	95 (339)	4.5 (16)	0.6 (2)	<0.001
	Sometimes	75.6 (99)	22.1 (29)	2.3 (3)	
	Often-Always	50 (35)	38.6 (27)	11.4 (8)	
3. Have you seen anybody being assaulted or beaten while being recorded and posted on the Internet?	Never	98 (445)	1.5 (7)	0.4 (2)	<0.001
	Sometimes	83.3 (50)	15 (9)	1.7 (1)	
	Often-Always	72.7 (32)	15.9 (7)	11.4 (5)	
4. Have you seen the dissemination of private photos or videos of anybody via mobile or Internet?	Never	97.1 (299)	2.3 (7)	0.6 (2)	<0.001
	Sometimes	87.6 (149)	10.6 (18)	1.8 (3)	
	Often-Always	65 (52)	18.8 (15)	16.3 (13)	
5. Have you seen photos being taken without somebody’s permission to be uploaded on the Internet or spread via phone?	Never	97.7 (387)	2 (8)	0.3 (1)	<0.001
	Sometimes	81.7 (85)	16.3 (17)	1.9 (2)	
	Often-Always	72.4 (42)	20.7 (12)	6.9 (4)	
6. Have you seen anonymous calls being made in order to scare someone or know someone who has received these types of calls?	Never	90.4 (293)	7.7 (25)	1.9 (6)	<0.001
	Sometimes	66.9 (95)	26.1 (37)	7 (10)	
	Often-Always	45.7 (42)	34.8 (32)	19.6 (18)	
7. Have you seen blackmail through calls or messages?	Never	93.4 (366)	5.4 (21)	1.3 (5)	<0.001
	Sometimes	75.9 (82)	22.2 (24)	1.9 (2)	
	Often-Always	58.6 (34)	20.7 (12)	20.7 (12)	
8. Have you seen or know of anyone who has been sexually harassed via mobile or Internet?	Never	97.3 (436)	2 (9)	0.7 (3)	<0.001
	Sometimes	83.9 (52)	11.3 (7)	4.8 (3)	
	Often-Always	60.4 (29)	14.6 (7)	25 (12)	
9. Have you known anybody who comments on other people’s blogs posting defamatory comments, lies, or secrets?	Never	96.7 (382)	2 (8)	1.3 (5)	<0.001
	Sometimes	87.4 (104)	11.8 (14)	0.8 (1)	
	Often-Always	72.7 (32)	13.6 (6)	13.6 (6)	
10. Do you know of anybody who has had their password stolen in order to prevent them from accessing their blog or email?	Never	95.4 (333)	3.2 (11)	1.4 (5)	<0.001
	Sometimes	89.4 (126)	8.5 (12)	2.1 (3)	
	Often-Always	66.2 (45)	17.6 (12)	16.2 (11)	
11 Have you seen edited photos or videos spread through social networks or YouTube to humiliate or laugh at some boy or girl?	Never	96.4 (375)	1.8 (7)	1.8 (7)	<0.001
	Sometimes	87.5 (98)	9.8 (11)	2.7 (3)	
	Often-Always	77.2 (44)	17.5 (10)	5.3 (3)	
12. Have you seen how someone has been harassed in order to try and isolate them from their contacts on social networks?	Never	96.8 (394)	2.2 (9)	1 (4)	<0.001
	Sometimes	87.1 (88)	9.9 (10)	3 (3)	
	Often-Always	50 (25)	26 (13)	24 (12)	
13. Have you seen how someone has been blackmailed into doing something that she/he did not want to do in exchange for not showing their intimate things on the Internet?	Never	97.5 (423)	1.8 (8)	0.7 (3)	<0.001
	Sometimes	81.4 (70)	14 (12)	4.7 (4)	
	Often-Always	63.2 (24)	21.1 (8)	15.8 (6)	
14. Do you know of anybody who has received threats regarding killing them or their family through a mobile phone, social network, or other technology?	Never	97 (458)	2.3 (11)	0.6 (3)	<0.001
	Sometimes	74.3 (55)	24.3 (18)	1.4 (1)	
	Often-Always	66.7 (8)	16.7 (2)	16.7 (2)	
15. Do you know anybody who has been defamed, by people lying on the Internet to sometimes discredit them or spread rumours to hurt them?	Never	91.7 (311)	7.1 (24)	1.2 (4)	<0.001
	Sometimes	77.7 (101)	19.2 (25)	3.1 (4)	
	Often-Always	47.2 (42)	36 (32)	16.9 (15)	

**Table 4:** Correlation of variables X-Y.

	CV	O	(1)	(2)	(3)	(4)	(5)	(6)	(7)	(8)	(9)	(10)
Cyber-victimisation (CV)	-	0.60 *	0.12 *	0.14 *	0.12 *	0.24 *	0.11 *	0.16 *	0.22 *	0.28 *	0.26 *	0.09 *
Observation (O)		-	0.14 *	0.23 *	0.24 *	0.08	0.06	0.18 *	0.27 *	0.27 *	0.29 *	0.23 *
Age (1)			-	0.18 *	0.06	−0.08	−0.04	0.08	0.16 *	0.13 *	0.20 *	0.16 *
Frequency of daily connection (2)				-	0.44 *	−0.15 *	0.02	0.19 *	0.15 *	0.08 *	0.12 *	0.14 *
Contacts on social network/messaging (3)					-	−0.08	0.04	0.15 *	0.13 *	0.09 *	0.10 *	0.12 *
Parental regulation and monitoring (4)						-	0.46 *	0.10 *	−0.01	0.08	0.03	−0.06
Parental co-use regulation (5)							-	0.08	−0.02	0.01	−0.06	−0.03
Emotional self-regulation-attention to emotions (6)								-	0.11 *	0.08	0.08	0.04
Attitude violence–As a form of fun (7)									-	0.76 *	0.77 *	0.62 *
Attitude violence–improvement of self-esteem (8)										-	0.79 *	0.53 *
Attitude violence–problem management (9)											-	0.63 *
Attitude violence-considered legitimate (10)												-

* The correlation is significant at the 0.05 level (bilateral).

**Table 5:** Wilks’ Lambda, using F-approximation (Rao’s F).

Function	Eigenvalue	%	Canonical Correlation	Wilk’s Lambda	F	df	*p*-Value
1 to 2	0.20	72.76	0.44	0.75	8.63	20,096	<0.001
2 to 2	0.07	27.24	0.27	0.93	4.79	9549	<0.001

**Table 6:** Correlations between original and canonical variables.

Scales	U_1_	U_2_
Cyber-victimisation (X11)	−0.89	0.46
Observation (X1)	−0.90	−0.43
Variables	V_1_	V_2_
Age (X3)	−0.34	−0.07
Frequency of daily connection (X4)	−0.48	−0.37
Contacts on social networks/messaging (X5)	−0.46	−0.48
Parental mediation. Parental monitoring (X73)	−0.39	0.67
Parental mediation. Co-use (X74)	−0.22	0.24
Emotional self-regulation. Subscale of attention to emotions (X81)	−0.43	−0.10
Violence. As a form of fun (X91)	−0.61	−0.22
Violence. Improvement of self-esteem (X92)	−0.68	0.05
Violence. Problem management (X93)	−0.68	−0.15
Violence. Considered legitimate (X94)	−0.41	−0.56

## Data Availability

The database is available to anyone who requires it, upon request addressed to the author of the correspondence.
